# Gynecological/Obstetric Background and Rheumatoid Arthritis: A Cross-sectional Study in Brazilian Patients

**DOI:** 10.1055/s-0041-1729149

**Published:** 2021-06-02

**Authors:** Anauá Fernanda dos Santos Cavalcante, Patrícia Martin, Thelma Larocca Skare

**Affiliations:** 1 Hospital Evangélico Mackenzie, Curitiba, PR, Brazil

**Keywords:** rheumatoid arthritis, pregnancies, menarche menopause, postmenopause, artrite reumatoide, gestação, menarca, menopausa, pós-menopausa

## Abstract

**Objective** To study a sample of rheumatoid arthritis (RA) patients for their gynecological/obstetric history and compare them to controls to determine their influences on number of pregnancies, menarche, menopause and reproductive years following RA onset.

**Methods** This is a cross-sectional study of 122 RA patients and 126 controls. Patients and controls were questioned about age of menarche, age of menopause, number of pregnancies and abortions. Reproductive years were calculated as the difference between age at menopause and age at menarche. For comparison, we used the Mann-Whitney, unpaired *t* , chi-squared, and Spearman tests. The adopted significance was 5%.

**Results** In the RA patients with disease beginning in the postmenopausal years, the period of reproductive years (age at menopause – age of menarche) showed a positive correlation with age at disease onset (rho = 0.46; 95% confidence interval [CI] = 0.20–0.55 with *p* = 0.0008). The number of pregnancies was higher in patients with postmenopausal disease onset when compared with those with premenopausal disease onset (median of 3 with interquartile range [IQR] = 2–4 versus median of 2 with IQR = 1–3; *p* = 0.009), and RA patients had more pregnancies than controls ( *p* = 0.0002).

**Conclusion** The present study shows that, in our population, the duration of reproductive years and the number of pregnancies are linked to the onset of RA.

## Resumo

**Objetivo** Estudar uma amostra de pacientes com artrite reumatoide (AR), com investigação da história ginecológica e obstétrica, comparando-a com controles, visando conhecer suas influências no número de gestações, menarcas, menopausa e anos reprodutivos no início da AR.

**Métodos** Trata-se de um estudo transversal de 122 pacientes com AR e 126 controles. Pacientes e controles foram questionados sobre idade da menarca, idade da menopausa, número de gestações e abortos. Os anos reprodutivos foram calculados com a diferença entre a idade da menopausa e a idade da menarca. Para comparação, foram utilizados Mann Whitney, Teste t não pareados, Teste qui-quadrado e teste de Spearman. A significância adotada foi de 5%.

**Resultados** Nas pacientes com AR e início da doença na pós-menopausa, o período de anos reprodutivos (idade da menopausa - idade da menarca) apresentou correlação positiva com a idade de início da doença (rho = 0,46; intervalo de confiança de 95% [IC95%] = 0,20–0,55 com *p* = 0,0008). O número de gestações foi maior nas pacientes com início da doença no período pós-menopausa quando comparadas às pacientes em pré-menopausa (mediana de 3 com intervalo interquartil [IIQ] = 2–4 versus mediana de 2 com IIQ = 1–3; *p* = 0,009). Nas pacientes com AR, foi observado um maior número de gestações do que no grupo controle ( *p* = 0,0002).

**Conclusão** O presente estudo mostra que, em nossa população, a diminuição dos anos reprodutivos e o alto número de gestações estão relacionados ao surgimento da AR.

## Introduction

Rheumatoid arthritis (RA) is the most common connective tissue disease, with a prevalence of 1% in the general population. 1 Similar to other connective tissue diseases, RA has a female predominance; the ratio ranges from 4 women to 1 man, when the disease begins in the reproductive years, to 2 to 1, when it initiates after 60 years. 2 3 Hormonal influences have been considered to play a role in this female preponderance as estrogens are considered to be agents with proinflammatory activity and capable of activating B cells. 2 4

Rheumatoid arthritis and female reproduction have been linked in the literature for decades, and the decrease in its symptoms during pregnancy is well recognized, with exacerbation of the disease in the postpartum period. 5 The gynecological and obstetrical history have been studied in this context; however, contradictory results have been obtained. According to an epidemiological investigation in a Swedish cohort, having more than one pregnancy amplified the risk of anti-citrullinated-protein antibody (ACPA)-negative RA in females of reproductive age. 6 Nevertheless, Guthrie et al. 7 studied 310 RA patients and 1,418 controls and found that parous women were around 40% less likely to receive the RA diagnosis. 6 7 Another study, by Peschken et al., 8 also showed that greater parity reduced the chances of being diagnosed with RA. In addition, a relationship between higher number of pregnancies and delayed onset of RA has been found. 7 9

Age at menopause has also been considered a risk factor in cases of postmenopausal RA, with data suggesting that early menopause increases the risk of this disease. 10 11 A Norwegian study evaluating 156 women with RA found reduced parity compared with the control group, suggesting decreased fertility in RA patients. 12

Rheumatoid arthritis is a disease with genetical and environmental factors that may combine with hormonal factors to influence the risk of developing it. 2 13 Therefore, conclusions on the features that can influence the onset of RA, such as gynecological and obstetrical history, may vary depending on the studied geographical region. Herein, we studied a sample of patients with RA, regarding their gynecological/obstetric history, and compared them to controls in order to determine the influence of the number of pregnancies, menarche, menopause, and reproductive years in the onset of RA, specifically in our region (south of Brazil).

## Methods

This is a cross-sectional study approved by the local Committee of Ethics in Research under protocol number 31727220.3.0000.0103 from the Mackenzie Presbyterian Institute; written consent was obtained from all patients. It studied 122 RA patients and 126 controls. To be included, RA patients had to fulfill at least 6 points in the American College of Rheumatology/European League Against Rheumatism (ACR/EULAR) classification criteria for RA. 1 14 Patients with disease onset prior to 16 years of age (juvenile form) or having any other associated inflammatory disease were excluded. This is a sample that encompasses all female patients with RA diagnosis from a single rheumatology unit from a university hospital that has a specialized rheumatoid arthritis outpatient clinic and that visited for regular consultations during the period of June to July 2020.

Patients and controls were questioned about the age of menarche, age of menopause, number of pregnancies, and number of abortions. Pregnancies and abortions were classified as appearing prior to or after RA onset. Reproductive years were calculated as the difference between age at menopause and age at menarche. The charts of RA patients were reviewed for clinical data (age of disease onset, and presence of RA clinical criteria) and serological data (rheumatoid factor). The control females were hospital employees and their relatives.

The results were analyzed with the help of the software Graph Pad Prism version 6.01 (Graph Pad Software, San Diego, CA, USA). To analyze the data distribution, the Shapiro-Wilk's test was used. The central tendency of parametric data was expressed in mean ± standard deviation (SD) and of non-parametric data as median and interquartile range (IQR). For comparison of numerical data (age, age of menarche, menopause, number of pregnancies, and number of abortions), we used the Mann-Whitney or unpaired *t* -tests. To compare nominal/categorical data (number of children/female and number of individuals at menopause), we used the chi-squared test. The correlation of the number of reproductive years with age at disease onset in RA patients with postmenopausal onset was done with the Spearman test. The adopted significance was 5%.

## Results

### The RA Studied Sample

The group of 122 RA patients had 290 children (2.3/patient). The description of the RA studied sample is on Table 1 .

**Table 1:** Description of the studied sample of rheumatoid arthritis

Variables	
Mean age (years) ± SD	57.2 ± 10.2
Median disease duration (years) (IQR)	9.5(6.0–17.0)
Mean age at diagnosis (years) ± SD	45.2 ± 12.0
RA beginning in post-menopause period (n)	49/121 (40.4%)
Median age at first son (years) (IQR)	22 (20–25)
Median pregnancies (n) (IQR)	3.0 (2.0–4.0)
One child	23/122 (18.8%)
Two children	32/122 (26.2%)
Three children	32/122 (26.2%)
Four children or more	35/122 (28.6%)
Median abortions (n) (IQR)	0 (0–0)
Positive rheumatoid factor (n)	78/122 (63.9%)

Abbreviations: IQR, interquartile range; n, number; SD, standard deviation.

In this sample, only 12 children from 12 mothers were born after the RA diagnosis had been made. No differences were found in the age of menarche, menopause, number of children and of abortions when RA patients with positive rheumatoid factor (RF) were compared with those with negative RF ( Table 2 ).

**Table 2:** Comparison of gynecological/obstetric background in rheumatoid arthritis patients according to the presence of rheumatoid factor

Variables	With positive RF N = 78	With negative RF N = 44	*p* -value
Median age at first pregnancy (IQR)	23.0 (19.0–26.0)	22.0 (20.0–25.0)	0.50
Disease onset prior to menopause	44/78 (56.4%)	28/44 (63.3%)	0.43
Median pregnancies (n) (IQR)	2.5 (2.0–4.0)	3.0 (2.0–3.7)	0.25
Median number of abortions	0 (0–0) 0–2	0 (0–0.75)	0.79

Abbreviations: IQR, interquartile range; n, number; RF, rheumatoid factor.

Table 3 shows the comparison of patients with pre and postmenopausal disease onset.

**Table 3:** Comparison of rheumatoid arthritis patients with disease onset prior and postmenopause

Variables	Fertile women N = 72	Women at menopause N = 50	*p* -value
Age at disease onset (n)	40 (31–45.7)	56 (50–60)	< 0.0001
Median age of menarche (years) (IQR)	13 (12–14)	13 (12–15)	0.57
Median pregnancies (n) (IQR)	2 (1 -3)	3 (2–4)	0.009
Number of abortions (n) IQR	0 (0–1)	0 (0–0)	0.96
Rheumatoid factor	44/72 (61.1%)	34/50 (68%)	0.43

Abbreviations: IQR, interquartile range; n, number.

In the subset of patients with RA that had postmenopausal disease onset , the period of reproductive years (age at menopause – age of menarche) showed a positive correlation with age at disease onset (rho = 0.46; 95% confidence interval [CI] = 0.20–0.55 with *p* = 0.0008).

### Comparison of Obstetrical and Gynecological History in RA and Controls

The comparison of these two samples is on Table 4 .

**Table 4:** Comparison of gynecological/obstetric history of rheumatoid arthritis patients with controls

Variables	RA patients N = 122	Controls N = 126	*p* -value
Median age (years) (IQR)	57.0 (51.0–64.2)	55.0 (50.0–60.5)	0.10
Postmenopausal females (n)	78/122 (63.9%)	81/126 (64.2%)	0.95
Median menarche age (years)	13.0 (12.0–14.7)	12.5 (12.0–14.0)	0.02
Median age at menopause (years (IQR)	48.0 (45.0–51.0)	50.0 (47.0–51.5)	0.02
Median pregnancies (n) (IQR)	2.0 (2.0–3.0)	2.0 (1.0–2.0)	0.0002
Pregnancies number/female			< 0.0001
Nulliparas (n)	0	12/125 (9.6%)	
One child (n)	26/122 (21.3%)	28/125 (22.4%)	
Two children (n)	40/122 (32.7%)	55/125 (44.0%)	
Three children (n)	30/122 (24.5%)	19/125 (15.2%)	
Four children or more (n)	26/122 (21.3%)	10/125 (8.0%)	
Median abortions (n) (IQR)	0 (0–0)	0 (0–1)	0.07

Abbreviations: IQR, interquartile range; n, number.

When the age of menopause of controls was compared with the age of menopause in RA patients with postmenopausal disease onset, we found that in controls the median age of menopause was 50 years (IQR = 47.0–51.5), while it was 46.0 (IQR = 42.7–50.0) in RA patients, with *p* = 0.003.

## Discussion

Our results have shown that RA patients had earlier menopause and later menarche than controls, suggesting that reproductive years in RA females are diminished. They also show that, if the number of reproductive years increases, the disease onset is delayed. These results point to a protective role of the length of reproductive years in the occurrence of this disease. Finding an early age for menopause in RA is consistent with previous results. A study with the Nurses' Health Study (NHS) cohort showed that early menopause increased the risk of RA (HR = 2.1). 11 Another study found that menopause onset at an age earlier than 40 years old more than doubled the risk of RA (odds ratio [OR] = 2.5). 15

Although estrogen is broadly considered to have a proinflammatory activity, its action is much more complex; this hormone may have diverse effects on the immune system. according to its concentration, on tissue receptor expression, and even on the female's reproductive stage. 2 Estrogens at periovular to pregnancy serum levels are capable of increasing B-cell responses, driving antibody secretion in healthy and autoimmune situations. 16 At similar levels, it stimulates the secretion of IL (interleukin) -4 and IL-10 and inhibits tumor necrosis factor (TNF) production, downregulating T cell-dependent immunity. 16 The secretion of IL-1B (a proinflammatory cytokine) by monocytes and macrophages is increased at periovulatory to early pregnancy levels; however, it is inhibited at late pregnancy levels. 16

Binding to the different receptors may also modulate the influence of estrogen on the immune system. Synovial cells of RA joints have both estrogen receptors, ER-α and ER-β, with higher density of ER-β, which is usually upregulated under hypoxic and inflammatory circumstances such as arthritis. 17 Studies in animal models have shown that the use of selective ER-β estrogens may have a repressive effect in the transcription of proinflammatory genes 16 ; this has led to the attempt to use them in the treatment of RA, unfortunately with negative results. 18

The number of pregnancies has been considered protective for RA by some authors but not by others. 7 8 10 In the present work, we found that RA patients have significantly more children than controls. In addition, the number of pregnancies was higher in patients with postmenopausal diagnosis. Altogether, these data suggest that the number of pregnancies is linked positively to this disease appearance. Nevertheless, it is important to remember that the premenopausal women are still fertile, and the number of pregnancies may increase, blurring this difference. Another point to pay attention to and that may have caused possible interpretation bias within this data are that we did not collect information on breast feeding. Although controversial, some authors found that long duration of breast feeding was associated with increased risk of developing RA. 17 19 20 Prolactin is considered both a hormone and a cytokine; it has an immune stimulatory effect, inhibiting the negative selection of autoreactive B lymphocytes and promoting autoimmunity. 21

The present work has several limitations: its cross-sectional design is one of them; another one is not having data on breast feeding. Also, it would have been interesting to analyze the influence of the gynecological/obstetric background according to the anti-citric citrullinated peptide (CCP) positivity. However, it does highlight the importance of menarche and menopause ages in the risk of developing RA, showing that more studies in this area could bring important information for the understanding and treatment of this disease.

## Conclusion

In conclusion, the present study shows that, in our population, the decrease in reproductive years and the high number of pregnancies are linked to the onset of RA.
